# Immune Checkpoint Inhibitor-Associated Cardiotoxicity: Current Understanding on Its Mechanism, Diagnosis and Management

**DOI:** 10.3389/fphar.2019.01350

**Published:** 2019-11-29

**Authors:** Yu-Wen Zhou, Ya-Juan Zhu, Man-Ni Wang, Yao Xie, Chao-Yue Chen, Tao Zhang, Fan Xia, Zhen-Yu Ding, Ji-Yan Liu

**Affiliations:** ^1^Department of Biotherapy, Cancer Center, and National Clinical Research Center for Geriatrics, West China Hospital of Sichuan University, Chengdu, China; ^2^Department of Dermatovenerology, West China Hospital, Sichuan University, Chengdu, China; ^3^Department of Neurosurgery, West China Hospital, Sichuan University, Chengdu, China; ^4^West China School of Medicine, Sichuan University, Chengdu, China

**Keywords:** immune checkpoint inhibitors, cardiotoxicity, myocarditis, pericarditis, cytotoxic T lymphocyte-associated antigen-4, programmed cell death protein 1, programmed cell death-ligand 1

## Abstract

Immune checkpoint inhibitors (ICIs) that target cytotoxic T lymphocyte antigen 4, programmed cell death-1, and PD-ligand 1 have revolutionized cancer treatment, achieving unprecedented efficacy in multiple malignancies. ICIs are increasingly being used in early cancer settings and in combination with various other types of therapies, including targeted therapy, radiotherapy, and chemotherapy. However, despite the excellent therapeutic effect of ICIs, these medications typically result in a broad spectrum of toxicity reactions, termed immune-related adverse events (irAEs). Of all irAEs, cardiotoxicity, uncommon but with high mortality, has not been well recognized. Herein, based on previous published reports and current evidence, we summarize the incidence, diagnosis, clinical manifestations, underlying mechanisms, treatments, and outcomes of ICI-associated cardiotoxicity and discuss possible management strategies. A better understanding of these characteristics is critical to managing patients with ICI-associated cardiotoxicity.

## Introduction

The immune system employs several suppressive molecules and pathways to maintain T lymphocyte cell tolerance and prevent autoimmunity (Boussiotis, 2016). Cytotoxic T lymphocyte-associated antigen-4 (CTLA-4), a coinhibitory molecule, is expressed on stimulated CD4^+^/CD8^+^ T cells to attenuate T cell activation. Of note, CTLA-4 also constitutively resides on Foxp3^+^ regulatory CD4^+^ T cells and directly facilitates the inhibitory function of regulatory T cells (Peggs et al., 2009; Walker and Sansom, 2011). Moreover, the programmed cell death protein 1 (PD-1)/programmed cell death-ligand 1 (PD-L1) pathway plays a predominant role in regulating T-cell-driven immune response. PD-1 exists inherently on the surface of T cells and is expressed on antigen-presenting cells (APCs), such as on macrophages and dendritic cells. The binding of these two molecules (PD1/PD/L1) can inhibit the immune response by reducing cytokine production and suppressing T-cell proliferation (Swaika et al., 2015). Intriguingly, numerous cancer cells overexpress PD-L1 on their surface, which contributes to their immune evasion by enhancing immune escape ability, resulting in a poor prognosis (Hino et al., 2010).

Based on the inhibitory roles of these checkpoint molecules or pathways, several immune checkpoint inhibitors (ICIs), including PD-1 inhibitors (nivolumab and pembrolizumab), PD-L1 inhibitors (atezolizumab, avelumab, and durvalumab), and CTLA-4 inhibitors (ipilimumab and tremelimumab) have been developed to restore the T cell-mediated immune response and improve the efficacy of anti-tumor treatments (Wolchok, 2015; Ribas and Wolchok, 2018). Encouragingly, these agents have revolutionized the treatment of various hematological and solid tumors (Powles et al., 2014; Tumeh et al., 2014; Pi et al., 2016).

The combination of ICIs, either use of multiple ICIs or ICIs combined with other therapies, such as chemotherapy, radiation, and anti-angiogenic drugs, has been associated with a significantly better prognosis than monotherapy (Larkin et al., 2015a). However, these reagents, both alone and in combination, also produce a wide spectrum of immune-related adverse events (irAEs), mainly due to aberrant autoreactive T cell activation (Weber et al., 2012; Nishino et al., 2015; Postow et al., 2018). Immune-mediated toxicities can affect any organ or tissue involving the skin, gastrointestinal system, endocrine system, lung, or liver (Champiat et al., 2016; Michot et al., 2016) and can largely be controlled by glucocorticoid therapy (Friedman et al., 2016). Among these toxicities, cardiotoxicity, a potentially fatal irAE, has rarely been reported in early clinical trials of ICI therapy because of its low incidence and nonspecific symptomatology (Mahmood et al., 2018a). Over the years, although increasing cases and case series of ICI-associated cardiotoxicity have been reported, it has not been fully recognized (Varricchi et al., 2017). Herein, to strengthen understanding of cardiotoxicity induced by ICIs and reduce deaths, we elaborate on the incidence, clinical manifestations, diagnosis, mechanisms, and outcomes of cardiotoxicity associated with ICIs. We will also discuss prophylactic strategies, potential treatments, and management of ICI-associated cardiotoxicity based on relevant literatures and current knowledge.

## Incidence and Clinical Manifestations of Immune Checkpoint Inhibitor-Associated Cardiotoxicity

**Figure 1:**
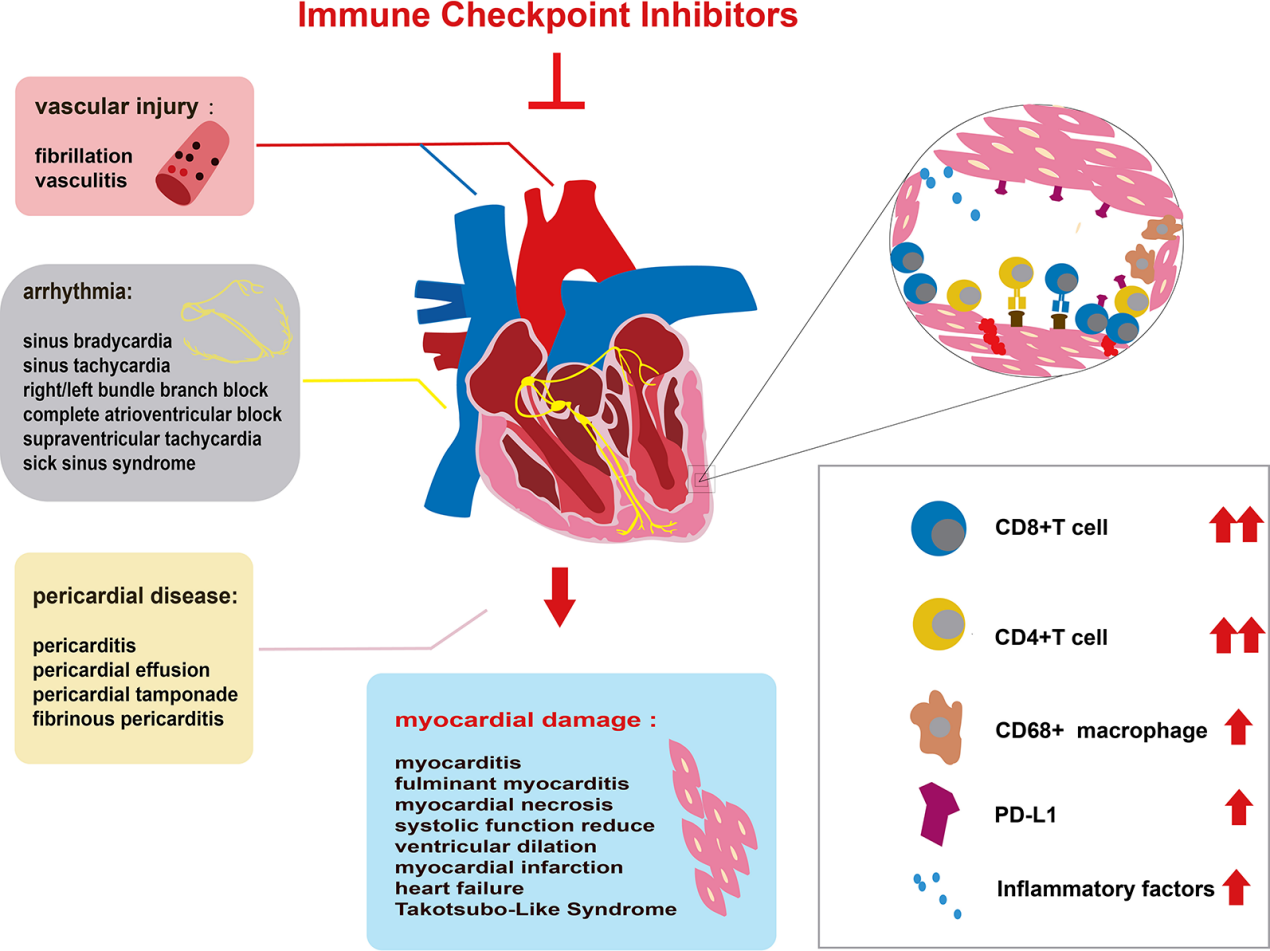
Immune checkpoint inhibitor (ICI)-related cardiac toxicity and its underlying mechanism. Anatomically, ICI-related cardiac toxicity involves almost all parts of the heart. The myocardium is most sensitive to ICI toxicity, showing impaired heart function. The cardiac conduction system, vascular system, and pericardium are also influenced by ICIs. Many infiltrating cells including hyperactivated CD4+/CD8+ T lymphocytes and a few macrophages (CD68+ cells) can microscopically be found in the heart tissue of patients with ICI-related cardiac toxicity. The infiltrating T cells are regarded as the main cause of ICI-related cardiac toxicity. The production of inflammatory factors promotes T cell activity. Elevated PD-L1 expression on cardiac muscle cells and T cells also contributes to the ICI-related cardiac toxicity.

Since the first specific case of ICI-associated cardiotoxicity was reported in 2014 (Heery et al., 2014), cardiotoxicity during ICI treatment has been reported in a gradually increasing number of patients (Geisler et al., 2015; Laubli et al., 2015; Heinzerling et al., 2016; Johnson et al., 2016a; Koelzer et al., 2016; Tadokoro et al., 2016; Behling et al., 2017; Norwood et al., 2017; Reuben et al., 2017; Tajmir-Riahi et al., 2018). Cardiotoxicity attributed to ICIs spreads to almost all parts of the heart (**Figure 1**) and involves both inflammatory cardiotoxicity and non-inflammation-mediated cardiotoxicity. The former includes myocarditis, perimyocarditis, pericarditis, left ventricular dysfunction without myocarditis, and others (Lyon et al., 2018). The latter includes asymptomatic noninflammatory left ventricular dysfunction (Roth et al., 2016), Takotsubo-like syndrome with both basal (Ederhy et al., 2018) and apical (Geisler et al., 2015; Anderson and Brooks, 2016) variants, coronary vasospasm (Nykl et al., 2017), arrhythmias (Salem et al., 2018), and myocardial infarction (Weinstock et al., 2017). Of all the cardiotoxicity-associated ICIs, myocarditis is the most common cardiotoxic reaction. Pericardial diseases and conduction diseases were reported in 15 and 12% of patients with ICI-related cardiotoxicity, respectively (Mir et al., 2018). One published study (Mahmood et al., 2018a) on 964 patients between 2013 and 2017 indicated that 1.14% of patients developed myocarditis and 0.52% developed a major adverse cardiovascular event (MACE), such as complete heart block, cardiogenic shock, and cardiac arrest, during treatment with ICIs. As for the presentation of myocarditis, it is highly variable and nonspecific (**Table 1**). Based on previously published studies, the manifestations of cardiotoxicity range from subclinical disease with asymptomatic cardiac biomarker elevation, fatigue, and general malaise to chest pain, dyspnea, palpitations, multiorgan failure, cardiogenic shock, and cardiac arrest (Laubli et al., 2015; Yun et al., 2015; Gibson et al., 2016; Escudier et al., 2017; Reuben et al., 2017; Mahmood et al., 2018a; Neilan et al., 2018; Wang et al., 2018; Samara et al., 2019).

**Table 1:** Published case reports and case series of immune checkpoint inhibitor-associated cardiotoxicity.

References	Patient	Medical history	Cancer type	Drug	Time of onset	Symptoms	Cardiotoxicity	Withdraw the drug	Treatment	Outcome
(Khoury et al., 2019)	68/M	Hypertension, Prostate cancer	Melanoma	Ipilimumab Nivolumab	2 weeks after second dose	Dyspnea, irregular heartbeats, achycardia	Myocarditis	YES	Solumedrol 1 g/day divided into four doses for 3 days. Prednisone 2 mg/kg and decreasing the dose daily by 7.5%.	Death
(Salem et al., 2019)	66/F	NR	Lung cancer	Nivolumab	Three doses	Chest pain	Myocarditis	NR	Methylprednisolone (50 mg/day) iv for 3 days, plasmapheresis, abatacept (500 mg q2w for five doses)	NR
(Esfahani et al., 2019)	71/F	NR	Melanoma	Pembrolizumab	Second cycle of treatment	Dyspnea	Myocarditis, Cardiac arrhythmia	NR	Methylprednisolone (1 g/day) iv for 3 days, then (2 mg/kg/day) mycophenolate mofetil (2 g/day), plasmapheresis, rituximab (375 mg/m^2^), alemtuzumab (30 mg)	NR
(Dhenin et al., 2019)	79/F	Asthma, Hypertension	Lung cancer	Pembrolizumab	After the third infusion	Chest pain	Pericarditis	Yes (Drug was reintroduced)	Pyridostigmine (30 mg, five times daily), methylprednisolone (80 mg/day).	Clinical recovery
(Altan et al., 2019)	72/M	Hypertension, CAD, smoking	Lung cancer	Anti-PD-L1	78 days	Dyspnea, hypotension hypoxia	Pericarditis	Yes	NR	Death
(Altan et al., 2019)	65/F	II-DM Hypertension, smoking	Lung cancer	Anti-CTLA-4, anti-PD-1	131 days	Loss of consciousness hypotension	Arrhythmias	Yes	Pacemaker	Death
(Altan et al., 2019)	57/M	Smoking	Lung cancer	anti-PDL1	98 days	Dyspnea, orthopnea, bilateral lower edema	Cardiac tamponade	Yes	NR	No additional toxicity after reintroduction
(Martin Huertas et al., 2019)	80/M	None	Kidney cancer	Nivolumab	After four cycles	Severe asthenia	Myocarditis, AF	Yes	Methylprednisolone (2 mg/kg/day IV)	Death
(Lindner et al., 2019)	73/M	PVD	UC	Pembrolizumab	After 22 cycles	Sweats, fatigue, fever, severe pain in the right limb	Vasculitis	Yes	NR	NR
(Fazel and Jedlowski, 2019)	78/F	Hypertension, Intermittent Asthma, PE, Depression	Melanoma	Nivolumab	5 days after the first cycle	Muscle weakness, dyspnea	Myocarditis	Yes	Methylprednisolone (1–1.5 mg/kg/day IV) to pulse steroid 1,000 mg/day IV, IGI (2 g/kg/day IV)	Deterioration
(Liu et al., 2019)	61/F	NR	Lung cancer	Atezolizumab	3 days after first dose of atezolizumab	Dyspnea, fatigue	Myocarditis	NR	Methylprednisolone 5 mg/kg/day IV, mycophenolate mofetil 1000 mg/day orally	Deterioration
(So et al., 2019)	55/F	A thymectomy for thymoma	Melanoma	Nivolumab	After the second infusion	Dysphagia, dyspnea, limb weakness	Myocarditis	Yes	IGI for four cycles, steroid pulse plus two cycles of plasma exchange	Symptoms improved
(Sakai et al., 2019)	74/M	NR	Lung cancer	Nivolumab	After the second infusion	General malaise, appetite decrease, dyspnea	MN	Yes	Large amount of catecholamine	Death
(Sharma et al., 2019)	76/F	Psoriatic arthritis	T-cell lymphoma	Brentuximab and Nivolumab	After the first infusion	Fatigue, dyspnea, orthopnea	AHF	Yes	Solumedrol 1 mg/kg for 3 days, Impella implantation.	Deterioration
(Charles et al., 2019)	33/M	NR	HL	Nivolumab	After the eight infusion	NR	CHB, Myocarditis	Yes	Mycophenolate mofetil, steroids (1 to 2 mg/kg), IGI	Death
(Agrawal et al., 2019)	73/M	NR	Malignant Mesothelioma;	Pembrolizumab;	32 days later	Progressive dyspnea, fatigue	Myocarditis	Yes	Prednisolone 60 mg/day orally, permanent pacemaker, IGI, plasmapheresis	Death
(Agrawal et al., 2019)	89/M	II-DM, Hypertension, Dyslipidemia, AF	Melanoma	Pembrolizumab	After the first dose	Weakness, myalgias, and dyspnea	Myocarditis	Yes	Methylprednisolone 1 g/day IV was started, then oral prednisone 60 mg twice daily, ATG	Death
(Agrawal et al., 2019)	65/F	Hypertension, MR	Lung cancer	Nivolumab	6 days later	Dyspnea, edema, bradycardia	ACS, ADHF	Yes	Methylprednisolone 1 g/day for 3 days, prednisone, furosemide, ATG	Deterioration
(Agrawal et al., 2019)	67/M	CAD	Melanoma	Nivolumab	Three cycles later	Chest pain, palpitations	Myocarditis	Yes	Prednisone 80 mg BID for 5 days then tapering, infliximab, oral corticosteroids	Symptoms improved
(Monge et al., 2018)	79/M	AF	Prostate cancer	Nivolumab	After 8 weeks	Blurred vision, pain, stiffness in the upper back	Myocarditis	Yes	Methylprednisolone 1 mg/kg/day and oral prednisone taper	Clinical recovery
(Hsu et al., 2018)	42/M	HBV carrier	HCC	Pembrolizumab	After six circles	Fatigue, dizziness and anorexia	Bradycardia	Yes	Cortisone 12.5 mg/day orally	Symptoms improved
(Gallegos et al., 2019)	47/F	CAD	Melanoma	Ipilimumab and Nivolumab, then Nivolumab	4 months	Dyspnea, achycardic, pulmonary edema	HF, ASVT	Yes	Methylprednisolone 500 mg intravenous BID for 5 days), infliximab (10 mg/kg/day for 2 days)	Death
(Thibault et al., 2018)	52/M	None	RCC	Nivolumab and Ipilimumab	Three circles later	None	Myocarditis	Yes (Nivolumab reintroduce)	Beta-blocker therapy	No subsequent clinical event
(Berner et al., 2018)	69/M	None	RCC	Avelumab and Axitinib	4 days after second dose	Fatigue, constipation	Hypertension, Cardiac arrest	Yes	Reduction of axitinib, amlodipine	Death
(Jain et al., 2018)	67/M	NR	Melanoma	Nivolumab and Ipilimumab	16 days after the first dose	Dyspnea, cough, dyspnea on exertion	ADHF, Arrhythmia, CHB	Yes	Methylprednisolone 500 mg twice daily, ATG and permanent pacemaker implanted	Deterioration
(De Almeida et al., 2018)	69/M	NR	Lung cancer	Nivolumab	5 days after the 24th cycle	Dyspnea, tachycardia, fever	Pericarditis, PT	Yes	Prednisone (1 mg/kg) for 2 weeks, gradually tapered for 8 weeks	Clinical recovery
(Ederhy et al., 2018)	45/F	NR	Melanoma	Nivolumab and Ipilimumab	5 days after the first infusion	NR	AHF, TLS	NR	Methylprednisolone, 1 g/day IV	Complete recovery
(Ederhy et al., 2018)	77/M	NR	Melanoma	Ipilimumab Nivolumab.	After 3 perfusions	NR	TLS	NR	Methylprednisolone 1 g/day IV for 3 days	NR
(Ganatra and Neilan, 2018)	41/F	Hashimoto’s thyroiditis	Melanoma	Ipilimumab and Nivolumab	6 days after four cycles	Dyspnea	Myocarditis	Yes	Methylprednisolone 1g/day for 3 days	Symptoms improved
(Yamaguchi et al., 2018)	60/M	None	Melanoma	Nivolumab	13 cycles later	Fatigue, fever	Fulminant Myocarditis	Yes	Prednisolone pulse therapy was initiated at 1000 mg/d for 3 days, IGI at 50 g/d for 2 days	Symptoms improved
(Mahmood et al., 2018b)	75/F	NR	EMC	Durvalumab and Tremelimumab	3 weeks after the first dose	Difficulty ambulating, dyspnea	Myocarditis, HF,CHB	Yes	Methylprednisolone 1 mg/kg, mycophenolate mofetil 1,000 mg oral twice daily	Symptoms improved
(Oristrell et al., 2018)	55/F	NR	Breast cancer	Pembrolizumab	Five cycles later	Pericardial chest pain	PT	Yes	Anterior pericardiectomy, corticosteroids 2 mg/kg/day IV and keep low doses	Symptoms improved
(Frigeri et al., 2018)	76/F	CD	Lung cancer	Nivolumab	After seven biweekly administrations of Nivolumab	Rapidly progressive dyspnea	Myocarditis, CAB	Yes	Methylprednisolone 5 mg/kg/d and three doses of infliximab 5 mg/kg	Deterioration
(Tajmir-Riahi et al., 2018)	72/M	NR	Melanoma	Nivolumab and Ipilimumab	After the 10th therapy	Dyspnea, edema of the legs	Myocarditis	Yes (Pembrolizumab reintroduce)	Prednisolone 1 mg/kg/day	Cardiacarrest
(Katsume et al., 2018)	73/M	Smoking	Lung cancer	Pembrolizumab	16 days after first dose	Faintness	CAB, Myocarditis	NR	Methylprednisolone, 1g/day IV for 3 days and temporary pacemaker implantation	NR
(Chen et al., 2018)	43/M	NR	Thymoma	Nivolumab	10 days later	Chest discomfort, fatigue, myalgias of lower limbs	Myocarditis	Yes	IGI 300 mg/kg IV for 4 days, methylprednisolone 1 g/day for 3 days followed by 500 mg/day for 4 days then 60 mg/day	Death
(Matson et al., 2018)	55/M	Hypertension, COPD	Lung cancer	Nivolumab	3 days after the second dose	Lethargy, dyspnea	ADRHF, cardiogenic shock	Yes	NR	Death
(Norwood et al., 2017)	49/F	Hyperlipidemia	Melanoma	Nivolumab and Ipilimumab	2 weeks after the first dose	Atypical chest discomfort at the cardiac apex	Myocarditis	Yes (following the Ipilimumab)	Methylprednisolone was initiated at 125 mg/day IV, IGI 400 mg/kg/day IV for 2 days	Clinical recovery
(Arangalage et al., 2017)	35/F	NR	Melanoma	Ipilimumab	15 days after the first infusion	Progressive dyspnea	Fulminant Myocarditis	Yes	Methylprednisolone, 1 g/day IV, and IGI IV, plasma exchanges	Completely recovered
(Penel et al., 2017)	61/M	Dyslipidemia, Smoking	Lung caner	Nivolumab	After the 11th dose	NR	ACS	Yes	Corticosteroids	Recovered
(Kimura et al., 2017)	54/M	NR	Lung cancer	Nivolumab	4 weeks after PD-1 therapy	Dizziness, nausea, loss of consciousness, general paralysis	HF	Yes	High-dose steroid, pacemaker	Death
(Behling et al., 2017)	63/M	Hypertension, Hyperlipoproteinemia, II-DM, COPD	Melanoma	Nivolumab	3 days after the second dose	Dyspnea, dysphagia, worsened muscle pain	AB, MI	Yes	Prednisone1.5 mg/kg IV and an antibiotic therapy with sultamicillin 3 g IV TID 500 mg aspirin and 5,000 IU unfractionated heparin	Death
(Johnson et al., 2016a)	65/F	NR	Melanoma	Nivolumab and Ipilimumab	12 days after the first doses	Atypical chest pain, dyspnea, fatigue	Fulminant myocarditis	Yes	Methylprednisolone 1 mg/kg/day IV	Death
(Johnson et al., 2016a)	63/M	NR	Melanoma	Nivolumab and Ipilimumab	15 days after the first doses	Fatigue, myalgias	Fulminant myocarditis	Yes	Methylprednisolone 1 g/kg/day IV for 4 days and infliximab	Death
(Roth et al., 2016)	60/M	Hypertension, anxiety, RS	Melanoma	Ipilimumab	2 years after the first dose.	None	AF	Yes	Lisinopril 5 mg/day, metoprolol was changed to carvedilol 6.25 mg twice daily	NR
(Tadokoro et al., 2016)	69/F	NR	Melanoma	Nivolumab	2 months	General malaise, palpitation	Myocarditis	Yes	Oral prednisolone (2 mg/kg) was initiated	Symptoms improved
(Heinzerling et al., 2016)	72/M	MI, II-DM, Hypertension, PVD, Hyperuricemia	Melanoma	Ipilimumab	After three infusions	Dyspnea, anasarca	Myocarditis	Yes	Corticosteroids were initiated at 1 mg/kg orally	Symptoms improved
(Heinzerling et al., 2016)	68/M	ADC, Alcohol abuse	Melanoma	Ipilimumab	After four doses	Dyspnea, lower extremity edema	Cardiomyopathy	Yes	Diuresis, coronary catheterization	Resolved
(Heinzerling et al., 2016)	71/M	None	Melanoma	Ipilimumab	After the second infusion	No obvious cardiac symptoms	MF	Yes	High dose steroids (2 mg/kg)	Death
(Heinzerling et al., 2016)	81/M	AF, CAD	Melanoma	Ipilimumab	11 weeks following the third dose	Progressive subacute dyspnea	HF, Myocarditis	Yes	Diuretics	Symptoms improved
(Heinzerling et al., 2016)	23/M	NR	Melanoma	Ipilimumab	7 months after initiating Ipilimumab	Chest pain and cough	Myocarditis/HF	Yes	Methylprednisolone (2 mg/kg/day) converted to 80 mg prednisone/day with taper over 1 month,	Resolved to baseline
(Heinzerling et al., 2016)	64/M	PVD	Melanoma	Ipilimumab	After the second dose	Fatigue, seizures, abdominal pain (Yun et al., 2015)	Myocarditis	Yes	Dopamine and fentanyl	Death
(Heinzerling et al., 2016)	88/M	CAD	Melanoma	Pembrolizumab	After the eight infusion	Myalgia, pain in the shoulder	Cardiac arrest	Yes	Corticosteroids 125 mg IV for 4 days	Resolved
(Heinzerling et al., 2016)	80/M	Melanoma	NHL	Ipilimumab	2 weeks after two doses	Dyspnea, edema, arrhythmias	Fatal myocarditis	Yes	Methylprednisolone (1 mg/kg) IV then prednisone 60 mg by mouth daily	Death
(Laubli et al., 2015)	73/F	NR	Melanoma	Pembrolizumab	Five cycles later	Progressive dyspnea	AHF	Yes	AT2-receptor blocker, a beta-blocker, spironolactone, diuretics	Symptomatic recovery
(Yun et al., 2015)	59/M	None	Melanoma	Ipilimumab	12 weeks after four cycles	Chest pain and dyspnea	AFP	Yes	Methylprednisolone 125 mg/day, prednisone 40mg/day, budesonide 9 mg/day on the third day, and tapered down over a month	Symptoms improved

NR, no report; HL, Hodgkin lymphoma; NHL, non-Hodgkin’s lymphoma; UC, urothelial carcinoma; HCC, hepatocellular carcinoma; RCC, renal cell carcinoma; EMC, endometrial cancer; AF, atrial fibrillation; MN, myocardial necrosis; HF, heart failure; AHF, acute heart failure; CHB, complete heart block; ACS, acute coronary syndrome; ADHF, acute decompensated heart failure; ASVT, asymptomatic supraventricular tachycardia; PT, pericardial tamponade; TLS, Takotsubo-like syndrome; CAB, complete atrioventricular block; ADRHF, acute decompensated right-sided heart failure; AB, atrioventricular block; MI, myocardial infarction; MF, myocardial fibrosis; AFP, acute fibrinous pericarditis; IV, intravenous; ATG, anti-thymocyte globulin; QD, once daily; BID, twice daily; TID, three times per day; II-DM, diabetes mellitus type II; RS, Raynaud syndrome; COPD, chronic obstructive pulmonary disease; CAD, coronary artery disease; PVD, peripheral vascular disease; AF, atrial flutter; MR, mitral regurgitation; ADC, asymptomatic dilated cardiomyopathy; CD, cardiovascular disease; PE, pulmonary embolism; mAb, monoclonal antibody; IGI, immunoglobulin.

As for the incidence and severity of cardiotoxicity, it is still not well recognized due to limited small-sample retrospective analyses and case reports regarding cardiotoxicity induced by ICIs. A multicenter retrospective study of 752 patients with melanoma treated with ipilimumab had shown that only one case of myocardial fibrosis had occurred (Voskens et al., 2013). Despite the lack of prospective randomized controlled trials to assess myocarditis, retrospective evaluation literature has estimated that the incidence of ICI-related myocarditis ranges from 0.09 (Johnson et al., 2016a) to 1.14% (Mahmood et al., 2018a). Although cardiotoxicity is rare, a high case fatality rate (35%) of myocarditis from ICIs had been reported in a systematic review by Hassan Mir (Mir et al., 2018). A systematic review by Wang et al. of fatal toxic effects associated with ICIs had found that ICI-related myocarditis appeared to present the highest (39.7%) death rate, with 52 deaths among 131 cases (Wang et al., 2018).

In patients treated with the combination of two ICIs, the incidence and death rate of cardiotoxicity is higher than with immunotherapy alone (Larkin et al., 2015b). The largest study of ICI-associated cardiotoxicity to date, using the global database (VigiBase) of the World Health Organization, has revealed that the incidence of myocarditis in patients treated with ICIs is 11 times greater than those without ICI treatment (Salem et al., 2018). A significantly higher case fatality rate (46%) in combination therapy was reported in this study. In addition, myocarditis was found to be more frequent (0.27 *vs.* 0.06%) and severe (60 *vs.* 10%) in patients prescribed a combination of nivolumab and ipilimumab than in those prescribed nivolumab alone (Johnson et al., 2016a). For the adverse events induced by the combination of nivolumab and ipilimumab, the rates of 1.3% for tachycardia, 1.1% for hypertension, 0.4% for arrhythmias, and 0.2% for atrial fibrillation were reported by the European Medicines Agency’s European public assessment report, Opdivo (Hassel et al., 2017). Nevertheless, when ICIs are combined with other non-ICI therapies, it remains unknown whether ICI-related myocarditis is more frequent. In a phase 1b trial of 55 patients treated with avelumab (anti-PD-L1 monoclonal antibody) plus axitinib (a vascular endothelial growth factor [VEGF] inhibitor), only one (1.8%) case developed lethal myocarditis (Choueiri et al., 2018).

**Figure 2:**
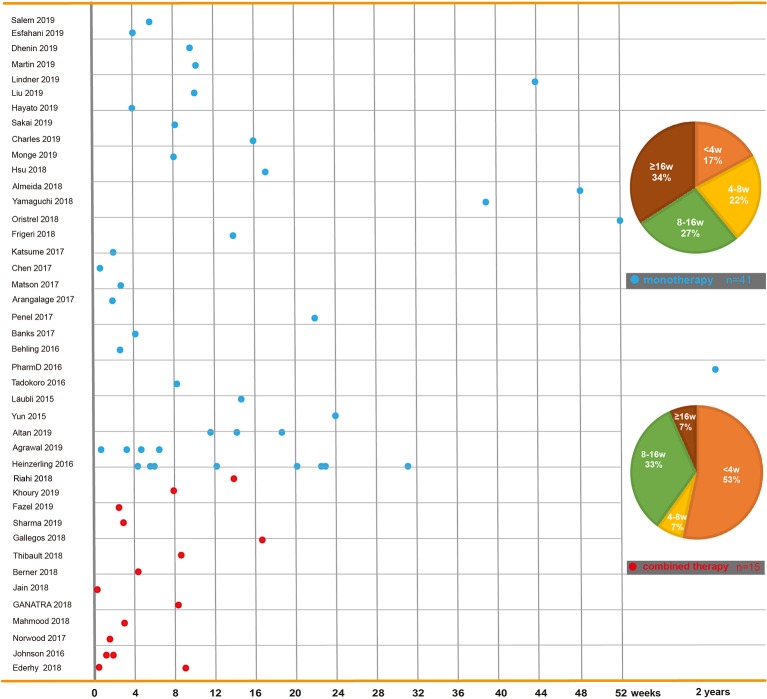
Time to onset of immune checkpoint inhibitor-related cardiac toxicity. In reported cases or case series, reagents including PD-1 inhibitors (nivolumab and pembrolizumab), PD-L1 inhibitors (atezolizumab, avelumab, and durvalumab), and CTLA-4 inhibitors (ipilimumab and tremelimumab) are used solely or in combination. This figure is built based on the cases’ therapy types (monotherapy and combined therapy). The time when the ICI-related cardiac toxicity occurred since the first dose of each case is recorded as a dot. The time to onset of monotherapy (the blue dot) and combined therapy (the red dot) are illustrated. The onset time trend of ICI-related cardiac toxicity in each group is shown. On the right, two pie charts reveal case percentages of different onset time periods in each group.

The time to onset of cardiotoxicity presentation varies depending on the medical history, type of medication, duration of usage, and double or single medication (**Table 1**). Approximately 80% of ICI-associated myocarditis occurs within the first 3 months of starting ICI therapy (Larkin et al., 2015a; Postow et al., 2015). Approximately 62–64% of patients received only one or two doses of ICIs before the onset of myocarditis (Moslehi et al., 2018; Atallah-Yunes et al., 2019). Cardiac disorders, including myocarditis, pericarditis, and cardiomyopathy are reported to occur between 2 and 17 weeks after ICI treatment onset (Wang et al., 2017; Oristrell et al., 2018). An analysis of an eight-center institutional registry indicated the median time for myocarditis was 34–65 days after initiation of treatment (Mahmood et al., 2018a). In contrast, a patient with melanoma had been reported to develop pericarditis 3 months after four cycles of ipilimumab (Yun et al., 2015). Interestingly, patients without any obvious symptoms had been found to have fulminant myocarditis after 1 year of ICI treatment (Yamaguchi et al., 2018). We reviewed previously published cases of adverse cardiac reactions and found that the onset time of cardiotoxicity was earlier in the combination of two ICIs. In the combination of two ICIs, in more than half (53%) of patients, cardiac toxicity occurred within 4 weeks after ICI initiation, whereas in ICIs alone, cardiac toxicity occurred in 17% of patients around the first ICI dose, and it occurred in 34% of patients 4 months later (**Figure 2**).

## Potential Mechanism of Immune Checkpoint Inhibitor-Related Cardiac Toxicity

The mechanism of ICI-related cardiac toxicity is not yet fully understood. Histological analyses of patients and monkey models with ICI-associated myocarditis have revealed that the infiltration of predominant CD4^+^/CD8^+^ T lymphocytes and a few macrophages (CD68^+^ cells) are the main cause of ICI-associated myocarditis (Johnson et al., 2016a; Ganatra and Neilan, 2018; Ji et al., 2019) (**Figure 1**). In addition, the expression change of multiple chemokine receptors further proves the enhancement of T cells. CXCR3–CXCL9/CXCL10 and CCR5/CCL5 are required for T cell activities to upregulate (Tokunaga et al., 2018; Ji et al., 2019). Tumor necrosis factor-α, granzyme B, and interferon-γ are produced by activated T cells, inducing cell death. These inflammatory molecules are overexpressed, which might contribute to cardiac injury (Varricchi et al., 2017; Tocchetti et al., 2018).

The most likely explanation is the “shared antigen” between the tumor and cardiac muscle, with muscle-specific antigens (desmin and troponin) detected in the tumor. Moreover, similar clonal T cell populations have been found infiltrating tumors and cardiac muscle. In this case, hyperproliferative T lymphocytes and macrophages aberrantly infiltrate the cardiac muscle after treatment with ICIs, thereby inducing fatal myocarditis (Johnson et al., 2016a). This theory is also supported by the shared epitope between myeloma cells and cardiomyocytes (Martinez-Calle et al., 2018).

Like tumor cells, cardiomyocytes might also employ the PD-1/PD-L1 and CTLA-4 pathways to prevent T cells from hyper-activation in physiological condition. ICIs, a promising anti-cancer agent, liberate the T-cell inhibition by tumor cells, may also relieve the same type of suppression by cardiomyocytes, which leads to T-cell hyperactivation in the heart. Subsequently, T-cell hyperactivation may result in ICI associated cardiotoxicity. CD28 binds to CD80 (B7-2)/CD86 (B7-2) on antigen-presenting cells (APCs) as a second activation signal to stimulate TCR signaling. CTLA-4 can complete with CD28 for binding with CD80/86 to inhibit the immune response. In contrast, silencing the genes that encode CTLA-4 promotes the proliferation and infiltration of CD8^+^ T cells in the heart, contributing to the development of myocarditis (Love et al., 2007). In many preclinical models, PD-1 has also been demonstrated to limit the T cell response in the heart as a negative immunoregulatory receptor. The disruption of PD-1 induces CD8^+^ T cell-mediated autoimmune dilated cardiomyopathy and myocarditis (Nishimura, 2001; Wang et al., 2010), suggesting PD-1 is protective against inflammation and cardiac damage (Tarrio et al., 2012).Similarly, the knockdown of PD-L1 also leads to mortal autoimmune myocarditis in a preclinical model of Murphy Roths Large mice (Lucas et al., 2008). Moreover, in patients with injured myocardium, PD-L1 is overexpressed on injured cardiomyocytes and infiltrating CD8^+^ T cells (Johnson et al., 2016a). Consistently, in preclinical studies of T cell-mediated myocarditis, the expression of PD-L1 is also upregulated (Grabie et al., 2007).The upregulation of PD-L1 might protect the myocardium from damage; however, this upregulation can be neutralized by ICIs (Grabie et al., 2007). In summary, these limited findings indicate that PD-1/PD-L1 and CTLA-4 play crucial roles in the emergence and development of ICI-related cardiac toxicity. Further studies are needed to elucidate the underlying mechanisms of these effects.

## The Diagnosis of Immune Checkpoint Inhibitor-Associated Cardiotoxicity

The detection of ICI-associated cardiotoxicity is currently challenging due to the lack of consistency of its clinical manifestation (**Table 1**). If ICI-related cardiotoxicity is observed in a patient, a detailed history and physical exams are required to exclude alternative cardiomyopathy etiologies, such as viral and autoimmune cardiac disease, infectious myocarditis or myocardial infarction. This comprehensive diagnostic information is helpful to correct circulation in a timely manner, to provide patients with specific treatment, and to help improve their symptoms (Catena et al., 2009).

**Figure 3:**
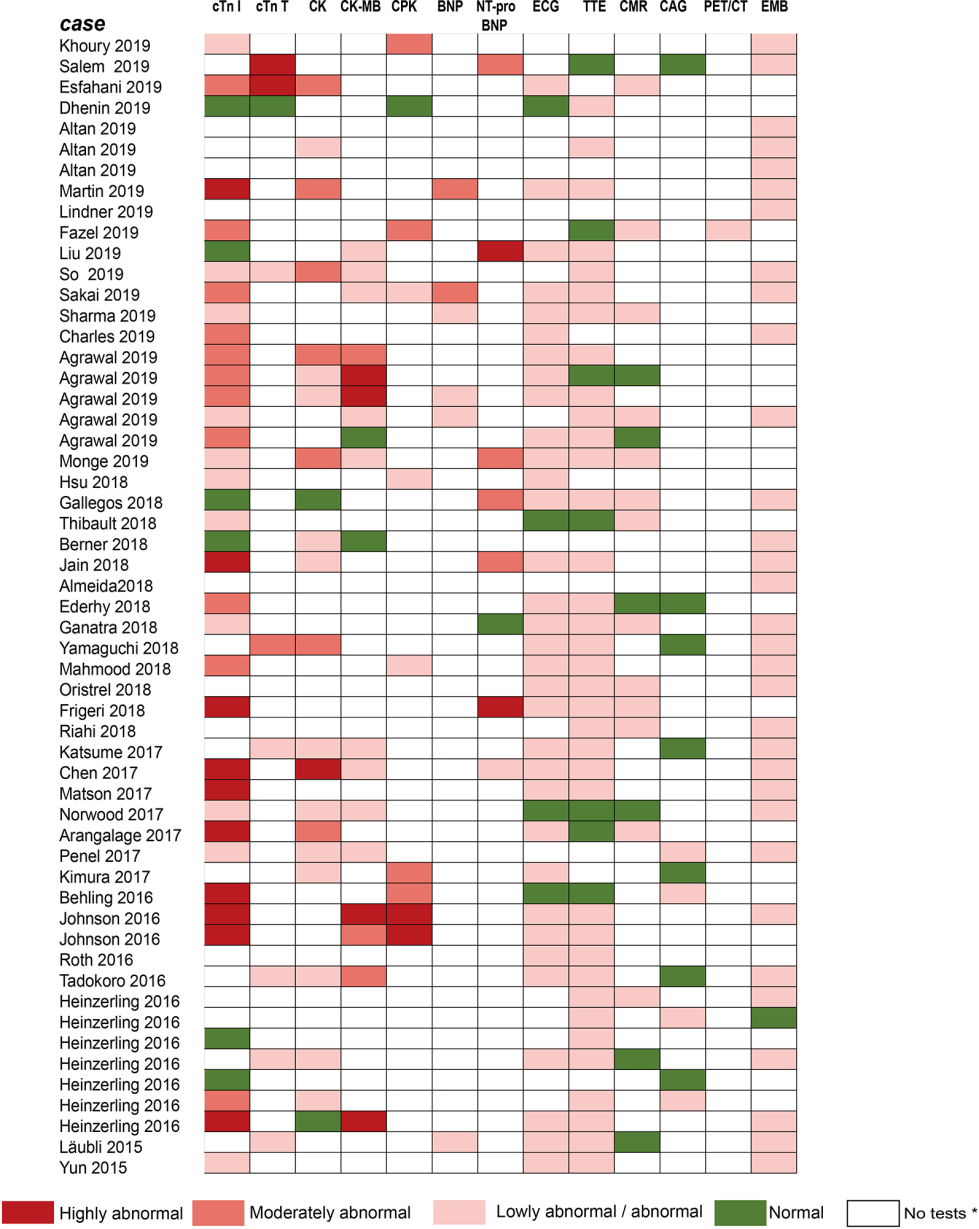
Summarized results of laboratory tests and other examinations for diagnosis in published cases reports/case series. The laboratory tests include cTnI, cTnT, CK, CK-MB, CPK, BNP, NT-pro-BNP. Other examinations include ECG, TTE, CMR, CAG, PET/CT, and EMB. No tests* means no laboratory testing or examination is performed in cases. Compared with the normal value of laboratory tests: Lowly abnormal: 10 times; Moderately abnormal: ≥10 and 100 times; Highly abnormal: ≥100 times. cTnI, cardiac troponin I; cTnT, cardiac troponin T; CPK, creatine phosphokinase; CK, creatine kinase; CK-MB, creatine kinase-myocardial band; BNP, brain natriuretic peptide; NT pro BNP, N-terminal pro-brain natriuretic peptide; ECG, electrocardiograph; TTE, transthoracic echocardiogram; CMR, cardiovascular magnetic resonance; CAG, coronary angiography; PET/CT, positron emission tomography/computed tomography; EMB, endomyocardial biopsy.

For early diagnosis of subclinical myocarditis, serial laboratory tests, electrocardiograms (ECGs) and transthoracic echocardiograms (TTEs) can be beneficial for patients treated with ICIs. Laboratory tests typically include troponin (cardiac troponin I [cTnI] or troponin T [cTnT]), creatine phosphokinase (CPK), creatine kinase (CK), creatine kinase-myocardial band (CK-MB), brain natriuretic peptide (BNP), and N-terminal pro-brain natriuretic peptide (NT-proBNP) (**Table 2**). Of all the markers, troponin is generally the most sensitive marker for confirming or excluding the diagnosis of myocarditis (Asnani, 2018). Mahmood et al. have indicated that almost all (94%) myocarditis cases had elevated troponin at the time of manifestation (Mahmood et al., 2018a). We reviewed the previous cases/case series and found that 84 and 89% of patients with ICI-associated cardiotoxicity had elevated cTnI and abnormal ECG, respectively (**Figure 3**). Even very low serum concentrations of troponin can provide important diagnostic and/or prognostic information for oncologists (Jaffe, 2011; Mahajan and Jarolim, 2011; Eggers and Lindahl, 2017). Notably, in patients with myositis, cTnT and CPK can also be elevated; thus, cTnI is the preferred marker for cardiac injury (Hughes et al., 2015). Interestingly, Mahmood et al. have also indicated that higher levels of serum cTnT might be associated with a greater risk of MACE (Mahmood et al., 2018a). BNP or NT-proBNP, the sensitive indicators (**Figure 3**), were reported to increase in most patients (Lyon et al., 2018); these are also helpful for the diagnosis of ICI-associated cardiotoxicity (Norwood et al., 2017). In cynomolgus monkeys with moderate mononuclear cell infiltration or with myocardial degeneration, an NT-proBNP or cTnI increase was observed at multiple time points after the first dose of ICIs; thus, they might serve as valuable biomarkers for ICI-induced myocarditis (Ji et al., 2019). However, BNP is a poorly specific marker for the diagnosis of ICI-associated cardiotoxicity, because it not only can be elevated in noninflammatory left ventricular dysfunction or other causes of acute cardiac injury, but also in many patients with cancer who have cardiotoxicity (Bando et al., 2017). The elevation of CK can also be observed in ICI-induced myocarditis (**Figure 3**) (Chen et al., 2018; Agrawal et al., 2019; So et al., 2019). Moreover, several reports have indicated that a complete atrioventricular block is usually associated with ICI-related myocarditis, with a considerably elevated CK level (Heinzerling et al., 2016; Johnson et al., 2016a). Of note, mild to massive elevation of serum CK was found in patients diagnosed with ICI-related myositis or myasthenia gravis (Kimura et al., 2016; Chang et al., 2017; Suzuki et al., 2017; March et al., 2018); therefore, the specificity of elevated CK was relatively poor.

**Table 2:** Laboratorial, radiological, and histopathological features of immune checkpoint inhibitor-associated cardiotoxicity.

Laboratory tests	ECG	TTE	CMR	Histopathological features
cTnI mildly elevated (a* = 9)	Sinus bradycardia (k* = 2)	LVSF diminished (Martin Huertas et al., 2019)	T2 intramyocardial intensity consistent with edema (Agrawal et al., 2019)	Inflammatoryinfiltrate beneath the thick fibrinous layeron the epicardium (CD4^+^, CD8^+^ T cells, some CD68^+^ macrophages, scattered CD20^+^ B cells (Altan et al., 2019)
cTnI moderately elevated (b* = 7)	Sinus tachycardia with ST-segment elevation in V1-6 (Sakai et al., 2019)	Reduced LVEF of 9% and akinesis of anteroseptal wall and apex (Sakai et al., 2019)	An elevated regional T2 ratio and EGE (Agrawal et al., 2019)	Myocardial necrosis (Martin Huertas et al., 2019)
cTnI massively elevated (c* = 8)	Sinus tachycardia with no ST-T changes (Jain et al., 2018; Sharma et al., 2019)	Severely reduced LVEF, moderate PE, moderate MR, severe TR and mildly RVD (Sharma et al., 2019)	A non-ischemic pattern of LGE, four-chamber dilation with severe biventricular dysfunction (Gallegos et al., 2019)	Intense inflammatory infiltrate: CD3^+^, CD8^+^, CD4^+^, 40% of all lymphocytes were PD-1 positive, some CD68^+^ macrophages (Martin Huertas et al., 2019)
cTnT elevated (Laubli et al., 2015; Yamaguchi et al., 2018)	Atrial rate was faster than ventricular rate (Charles et al., 2019)	Mild concentric LVH, mild RAE, moderate LAE, and mild AR, MR, TR (Agrawal et al., 2019)	Patches of LGE were seen in the basal and mid inferior wall showing an epicardial pattern compatible with myocarditis (Monge et al., 2018)	Myocardial necrosis with few inflammatory cells scattered in both ventricles (Sakai et al., 2019)
cTnI decreased (Liu et al., 2019)	Alternating RBBB and LBBB, episodes of asystole, third-degree block with a junctional escape rhythm (Agrawal et al., 2019)	RVD (Agrawal et al., 2019)	Diffuse myocardial edema (Ederhy et al., 2018)	Intense inflammatory infiltrate: CD4^+^, CD8^+^ T cells. PD-L1 stain showed focal membrane positivity in the areas of LGE (Gallegos et al., 2019)
CK mildly elevated (Katsume et al., 2018)	RBBB (Agrawal et al., 2019)	EF was severely decreased to 25–30% (Agrawal et al., 2019)		Lymphocytic infiltrate: CD3^+^, CD4^+^, CD8^+^ T cells, CD68^+^ macrophage within the myocardium, cardiac sinus and atrioventricular nodes (Johnson et al., 2016a)
CK moderately elevated (d* = 7)	Sinus rhythm with new lateral ST segment depressions (Agrawal et al., 2019)	Mild BVD with reduced RVSF, BAD (Agrawal et al., 2019)		T-cell and macrophage infiltrates in the myocardium, cardiac conduction system and skeletal muscle (Johnson et al., 2016a)
CK massively elevated (Chen et al., 2018; Agrawal et al., 2019)	Atrial tachycardia (Gallegos et al., 2019)	GBF with LVEF of 26%, severe LVD (Fazel and Jedlowski, 2019), and a trivial PE (Gallegos et al., 2019)		Heavy infiltration of CD68^+^ and CD3^+^, CD20^-^ T-lymphocytes (Berner et al., 2018)
CPK mildly elevated (Sakai et al., 2019)	PR prolongation with normal QRS complexes (Johnson et al., 2016a)	LVEF of 65% with LAE, RVD and increased PAP (Johnson et al., 2016a)		Diffuse cardiomyocyte necrosis with lymphocytic infiltration and predominance of CD3^+^ and CD20^-^ T cells (Jain et al., 2018)
CPK moderately elevated (e* = 3)	Profound ST segment depression (Johnson et al., 2016a)	Severe LV hypokinesis and LVEF decline to 20% (Jain et al., 2018).		Nonspecific chronic inflammation with extensive fibrosis and lymphocyte infiltration (De Almeida et al., 2018)
CPK massively elevated (Johnson et al., 2016a; Katsume et al., 2018)	AF with QT prolongation and LAFB (Monge et al., 2018)	Restrictive PE (De Almeida et al., 2018)		Diffuse infiltration with inflammatory cells (histocytes, lymphocytes, macrophages, and giant cells) with cardiac myocyte necrosis (Mir et al., 2018)
CK-MB mildly elevated (f* = 5)	Complete atrioventricular block with wide QRS complexes (Katsume et al., 2018)	Reduced LVEF (40%) with apical and mid-ventricular akinesia (Ederhy et al., 2018)		Lymphocytic infiltration: CD3^+^, CD4^+^, CD8^+^ CD20^-^, strong expression of PD-L1 (Chen et al., 2018)
CK-MB moderately elevated (g* = 3)	Intraventricular conduction delay progressed into episodes of ventricular tachycardia (Jain et al., 2018)	GlobalLV systolic dysfunction with an EF of 15% (Ganatra and Neilan, 2018)		Extensive lymphocytic infiltration, interstitial edema, and myocardial necrosis and with predominant CD4^+^, CD8^+^, CD20^-^, PD-L1 strongly expressed on myocardium (Yamaguchi et al., 2018)
CK-MB massively elevated (Johnson et al., 2016a)	Sinus tachycardia with T-wave inversion in the anteroseptal leads (Ederhy et al., 2018)	Thickened interventricular septum (12 mm), regular ventricular motion with LVEF of 49% (Chen et al., 2018)		Lymphocytic infiltration with occasional eosinophils (Mahmood et al., 2018b)
BNP mildly elevated (h = 5*)	T-wave inversion on leads V2, V3, and V4 (Ederhy et al., 2018)	Diffuse hypokinesis and reduced LVEF (15%) with myocardial edema (Yamaguchi et al., 2018)		Diffuse lymphoplasmacytic infiltrates (CD3^+^, CD4^+^, CD8^+^, CD20^-^ cells) with foci of active myocyte injury and necrosis throughout the atria, ventricles, and interventricular septum, (Matson et al., 2018)
BNP moderately elevated (i = 4*)	Low QRS voltage and T wave inversion on V1–V4 leads (Yun et al., 2015)	54% LVEF with regional areas of hypokinesis (Mahmood et al., 2018b)		CD3^+^ infiltrated in the pericardium; huge infiltration in pericardium with predominance of neutrophils (Oristrell et al., 2018)
BNP massively elevated (Agrawal et al., 2019)	Sinus rhythm with prolongation of the PR interval and RBBB (Chen et al., 2018)	RVD with reflux into the hepatic veins, suggestive of RHF (Matson et al., 2018).		Interstitial fibrosis with inflammation, fiber necrosis, signs of hypertrophy (Tajmir-Riahi et al., 2018)
NT-pro BNP elevated (j = 5*)	ST segment Elevation in V4–V6, leads II, III, and aVF (Yamaguchi et al., 2018)	Moderate PE and right atrial systolic collapse (Oristrell et al., 2018)		Early collagen deposition admixed with inflammatory cells; the majority of CD3^+^, CD4^+^, CD68^+^; the rarity of CD20^+^, CD138 (Norwood et al., 2017)
CRP mildly elevated (Katsume et al., 2018; Fazel and Jedlowski, 2019)	CAB (Kimura et al., 2017; Mahmood et al., 2018b)	A severely reduced LVEF, MAB (Frigeri et al., 2018)		Lymphocytic infiltration: CD3^+^, CD8^+^ cells with the myocardium (Johnson et al., 2016a)
CRP massively elevated (Martin Huertas et al., 2019)	Sinus tachycardia (Oristrell et al., 2018)	Diffuse hypokinesis of the LVEF (30.2%) (Katano et al., 2011)		Lymphocytic infiltration with a predominance of CD8+ T cells (Katano et al., 2011)
AChR Ab mildly elevated (Martin Huertas et al., 2019)	Sinus tachycardia with a RBBB and ST-segment elevation in the anteroseptal and inferolateral leads (Arangalage et al., 2017)	A severely impaired LVEF of 30% with marked ventricular desynchrony (Laubli et al., 2015)		Patchy lymphocytic infiltration: CD3^+^, CD8^+^, CD68^+^ cells (Heinzerling et al., 2016)
AChR Ab massively elevated (So et al., 2019)	ST-segment elevation in leads II, III, and aVF (Katano et al., 2011)			Lymphocytic infiltration with a predominance of CD8^+^ T cells (Laubli et al., 2015)
	A suspected non-ST segment elevation MI (Behling et al., 2017)			Mixed inflammatory infiltrates in the pericardial wall, accompanied by abundant surface fibrin (Yun et al., 2015)
	PR interval prolongation with normal QRS complexes; rapid progression to CHB (Johnson et al., 2016a)			
	Tachycardiac sinus rhythm with ventricular bigamy (Laubli et al., 2015)			

Compared with the normal value of laboratory tests: mildly elevated: < 10 times; moderately elevated: ≥10 and <100 times; massively elevated: ≥100 times. cTnI, cardiac troponin I; cTnT, cardiac troponin T; BNP, brain natriuretic peptide; TTE, transthoracic echocardiogram; ECG, electrocardiogram; CMR, cardiovascular magnetic resonance; CK-MB, creatine kinase-myocardial band; NT-pro BNP, N-terminal pro-brain natriuretic peptide; CPK, creatine phosphokinase; CK, creatine kinase; CRP, C-reactive protein; AChR Ab, acetylcholine receptor antibody; EF, ejection fraction; LV, left ventricular; LVEF, left ventricular ejection fraction; PE, pericardial effusion; MR, mitral regurgitation; AR, aortic regurgitation; BVD, bi-ventricular dilatation; BAD, bi-atrial dilatation; LVD, left ventricle dilatation; RVD, right ventricle dilatation; TR, tricuspid regurgitation; LAFB, left anterior fascicular block; LBBB, left bundle branch block; RBBB, right bundle branch block; LVH, left ventricular hypertrophy; RAE, right atrial enlargement; LAE, left atrial enlargement; GBF, global biventricular failure; LGE, late gadolinium enhancement; EGE, early gadolinium enhancement AF, atrial fibrillation; PAP, pulmonary artery pressure; LVSF, left ventricular systolic function; RVSF, right ventricular systolic function; RHF, right heart failure; CHB, complete heart block; MI, myocardial infarction; MAB, multiple apical thrombi; CAB, complete atrioventricular block

(a* = 9 (Norwood et al., 2017; Ganatra and Neilan, 2018; Katsume et al., 2018; Monge et al., 2018; Thibault et al., 2018; Agrawal et al., 2019; Charles et al., 2019; Sakai et al., 2019; Sharma et al., 2019).

(b* = 7) (Ederhy et al., 2018; Frigeri et al., 2018; Matson et al., 2018; Agrawal et al., 2019; Charles et al., 2019; Fazel and Jedlowski, 2019; Khoury et al., 2019).

(c* = 8) (Johnson et al., 2016a; Arangalage et al., 2017; Chen et al., 2018; Martin Huertas et al., 2019; Salem et al., 2019).

(d* = 7) (Arangalage et al., 2017; Monge et al., 2018; Yamaguchi et al., 2018; Agrawal et al., 2019; Martin Huertas et al., 2019; Salem et al., 2019; So et al., 2019).

(e* = 3) (Behling et al., 2017; Kimura et al., 2017; Khoury et al., 2019).

(f* = 5) (Norwood et al., 2017; Chen et al., 2018; Katsume et al., 2018; Liu et al., 2019; Sakai et al., 2019).

(g* = 3) (Johnson et al., 2016a; Monge et al., 2018; Agrawal et al., 2019).

(h* = 5) (Laubli et al., 2015; Monge et al., 2018; Agrawal et al., 2019; Fazel and Jedlowski, 2019; Sharma et al., 2019).

(i* = 4) (Gallegos et al., 2019; Liu et al., 2019; Martin Huertas et al., 2019; Sakai et al., 2019).

(j* = 5) (Chen et al., 2018; Frigeri et al., 2018; Ganatra and Neilan, 2018; Liu et al., 2019; Salem et al., 2019).

(k* = 2) (Hsu et al., 2018; Liu et al., 2019).

Given ECG has widespread availability and is easy to perform, it is considered a first-line test to identify patients with suspected ICI-associated myocarditis (Ganatra and Neilan, 2018). In our review of previous case reports, most patients had had an ECG performed (**Figure 3**). Abnormal ECGs have been reported in 40–89% of patients with ICI-related cardiotoxicity; however, these changes are often nonspecific (**Table 2**) (Escudier et al., 2017; Mahmood et al., 2018a). TTE has also been applied to provide further insight into left ventricular ejection fraction (LVEF) impairment, pericardial effusions, and wall motion abnormalities. However, a normal TTE report does not rule out ICI-associated myocarditis (Neilan et al., 2018; Tocchetti et al., 2018). Patients presenting with cardiac marker elevation, ST elevation, and ischemic symptoms should receive emergency coronary angiography to eliminate acute coronary syndrome (Tajiri et al., 2018).

To accurately diagnose myocarditis, further diagnostic technologies, such as cardiac magnetic resonance (CMR) imaging or an endomyocardial biopsy (EMB) are also necessary (Guglin and Nallamshetty, 2012; Bami et al., 2016). Gadolinium contrast-enhanced CMR imaging, a meaningful noninvasive diagnostic tool superior to echocardiography, can identify tissue characterization and offer accurate diagnosis of fibrosis and inflammation for hemodynamically stable patients in the early course of the disease (Friedrich et al., 2009; Aquaro et al., 2017). The CMR features of myocarditis, including edema, necrosis, and scar formation, were previously defined as the Lake Louise Criteria (**Table 2**) (Friedrich et al., 2009). For instance, an enhanced T2 signal on CMR could be indicative of underlying myocardial edema or myocarditis (Sharma et al., 2019). However, for patients who require invasive hemodynamic or respiratory and/or circulatory support, CMR imaging might not be feasible. Furthermore, although CMR imaging to detect myocardial edema and late gadolinium enhancement is accurate, its sensitivity is relatively poor (Laubli et al., 2015; Escudier et al., 2017; Norwood et al., 2017; Mahmood et al., 2018a) and an absence of positive findings on CMR does not rule out myocarditis (Abdel-Aty et al., 2005).

EMB, a gold standard for the diagnosis of myocarditis (Kindermann et al., 2012; Leone et al., 2012; Caforio et al., 2013), should be conducted when the treatment course is affected by suspected cardiotoxicity. This is especially true in unclear situations in which the oncologist does not know whether to continue or terminate ICI treatment. An EMB can reveal various features of interstitial inflammation suggested by interstitial fibrosis and lymphocyte infiltration (Sharma et al., 2019). Previous reports on ICI-related myocarditis have shown that significant T cells (CD4^+^, CD8^+^) and macrophage infiltration were observed in the myocardium (Laubli et al., 2015; Heinzerling et al., 2016; Johnson et al., 2016a; Koelzer et al., 2016; Tadokoro et al., 2016), cardiac conduction system (Johnson et al., 2016a), interventricular septum (Matson et al., 2018) and pericardium (Oristrell et al., 2018) (**Table 2**). Similar T cell populations were also observed in cardiomyocytes, according to a postmortem report of a patient who died from ICI-induced myocarditis (Johnson et al., 2016a), suggesting hyperactivated cytotoxic T cells directly injuring themyocardium as the probable mechanism of myocarditis induced by ICIs. B lymphocytes and/or plasma cells are usually rare or absent (Altan et al., 2019). Inflammatory infiltration can be transient and focal, however, and can sometimes be inaccessible to pathological puncture. Therefore, a biopsy sampling error from patients with myocarditis can result in a false negative diagnosis (Leone et al., 2012). In this case, it is suggested that EMB should be reattempted in cases with unexplained progressive heart failure (Caforio et al., 2013).

## Treatment and Outcome of Immune Checkpoint Inhibitor-Associated Cardiotoxicity

The treatment regimens for ICI-associated cardiotoxicity vary depending on the case (**Table 1**); however, the principal strategy concentrates on targeting the hyperactive T-cell response. High-dose steroids have constituted the first-line treatment for ICI-related myocarditis (Heinzerling et al., 2016; Johnson et al., 2016a; Kimura et al., 2016; Semper et al., 2016; Tadokoro et al., 2016; Haanen et al., 2017; Brahmer et al., 2018). Prompt initiation of high-dose intravenous methylprednisolone and immediate ICI discontinuation are associated with improved symptoms (Escudier et al., 2017; Mahmood et al., 2018a). A higher starting dose of steroids (intravenous methylprednisolone 1 g) was related to a lower rate of MACE according to a recent study by Mahmood et al. (Mahmood et al., 2018a). Although data regarding treatment for irAEs from rigorous studies are lacking, rapidly initiating intravenous or oral prednisone (1–2 mg/kg) for most patients and intravenous methylprednisolone (0.5–1.0g) for refractory cases with progressive tapering are recommended according to consensus guidelines (Brahmer et al., 2018). However, multiple studies have indicated that corticosteroids alone might not be sufficient to improve immune-mediated cardiac adverse reactions, and patients with ICI-associated cardiac events might even progress to malignant arrhythmias and severe heart failure symptoms during steroid treatment (Heinzerling et al., 2016; Johnson et al., 2016a). Many patients had received corticosteroids early in their cardiotoxicity management, tapering them over 1 month; however, no significant effect was observed (Norwood et al., 2017; Agrawal et al., 2019; Martin Huertas et al., 2019).

For patients with a poor response to corticosteroids, other immunosuppressive drugs should be administered, including immunoglobulin (Caforio et al., 2013), plasmapheresis, mycophenolate mofetil, tacrolimus, and infliximab (Haanen et al., 2017; Jain et al., 2017; Norwood et al., 2017; Reddy et al., 2017; Brahmer et al., 2018; Frigeri et al., 2018; Lyon et al., 2018). Infliximab, a chimeric immunoglobulin G1 monoclonal antibody blocking tumor necrosis factor-α, is used to treat patients with steroid-refractory ICI-associated colitis (Pages et al., 2013). The use of infliximab has been documented in the context of severe steroid-refractory myocarditis (Heinzerling et al., 2016; Johnson et al., 2016a; Tay et al., 2017; Frigeri et al., 2018) and has demonstrated significant clinical recovery and biochemical normalization (Agrawal et al., 2019). It is cautioned that infliximab could be potentially associated with deteriorating heart failure and is prohibited for patients with moderate to severe heart failure (Kwon et al., 2003). Considering the histological similarity between ICI-associated myocarditis and cardiac transplantation rejection, anti-transplant rejection medications (e.g., anti-thymocyte globulin [ATG]) have also been used for treating patients with ICI-related myocarditis (Tay et al., 2017). One case series has indicated that two patients treated with ATG after their clinical course worsened during steroid treatment responded well to ATG therapy, with remission of cardiogenic shock and malignant arrhythmias (Agrawal et al., 2019). The underlying mechanism could be associated with ATG leading to a rapid reduction in lymphocyte infiltration and T cell superactivation, thereby resulting in myocardial conduction improvement (Tay et al., 2017).

Recently, two reports (Esfahani et al., 2019; Salem et al., 2019) have shown that the new therapeutic agents alemtuzumab and CTLA-4 agonists (abatacept and belatacept), could be associated with significant relief of symptoms of cardiotoxic reactions caused by ICIs. Alemtuzumab, a monoclonal antibody that binds to CD52, can result in destruction of complement-mediated peripheral immune cells (monocytes, lymphocytes, macrophages, natural killer cells, and dendritic cells). Although the use of alemtuzumab in the context of cardiac allograft rejection has previously been evaluated, data on its use in patients with irAEs is limited (Cahoon et al., 2012). A recent report has indicated that 30 mg of alemtuzumab led to rapid T-cell depletion and was associated with the resolution of cardiac immunotoxic effects (Esfahani et al., 2019). CTLA-4 agonists, either abatacept or belatacept, can inhibit T cell costimulation mediated by CD28/B7 at the dendritic cell level, thereby abrogating the costimulation of T cells upstream of PD-1/PD-L1 and the CTLA-4 pathways. Abatacept can rapidly cause global T cell anergy (the inactivation of normal immune response) with specific reverse pathways activated by ICIs (Ingelfinger and Schwartz, 2005). When high-dose methylprednisolone injection and sustained plasmapheresis did not work, abatacept resulted in a rapid reduction in cTnI levels and recovery of LVEF (Salem et al., 2019). However, given the potential risks of infectious complications and tumor growth, it is necessary to further evaluate of the risk–benefit balance of abatacept in ICI-induced myocarditis (Ingelfinger and Schwartz, 2005).

Apart from the immunosuppressive therapies above, when necessary, beta-blockers, angiotensin converting enzyme inhibitors (ACEIs), and high-dose aspirin could be required as auxiliary therapies for patients with heart failure and in the context of raised troponin and the indication of cardiac ischemia (Berner et al., 2018). Extracorporeal membrane oxygenation is also required for a patient with severe myocarditis induced by the combination therapy of nivolumab and ipilimumab (Arangalage et al., 2017).

## Possible Management of Immune Checkpoint Inhibitor-Associated Cardiotoxicity and Future Directions

**Figure 4:**
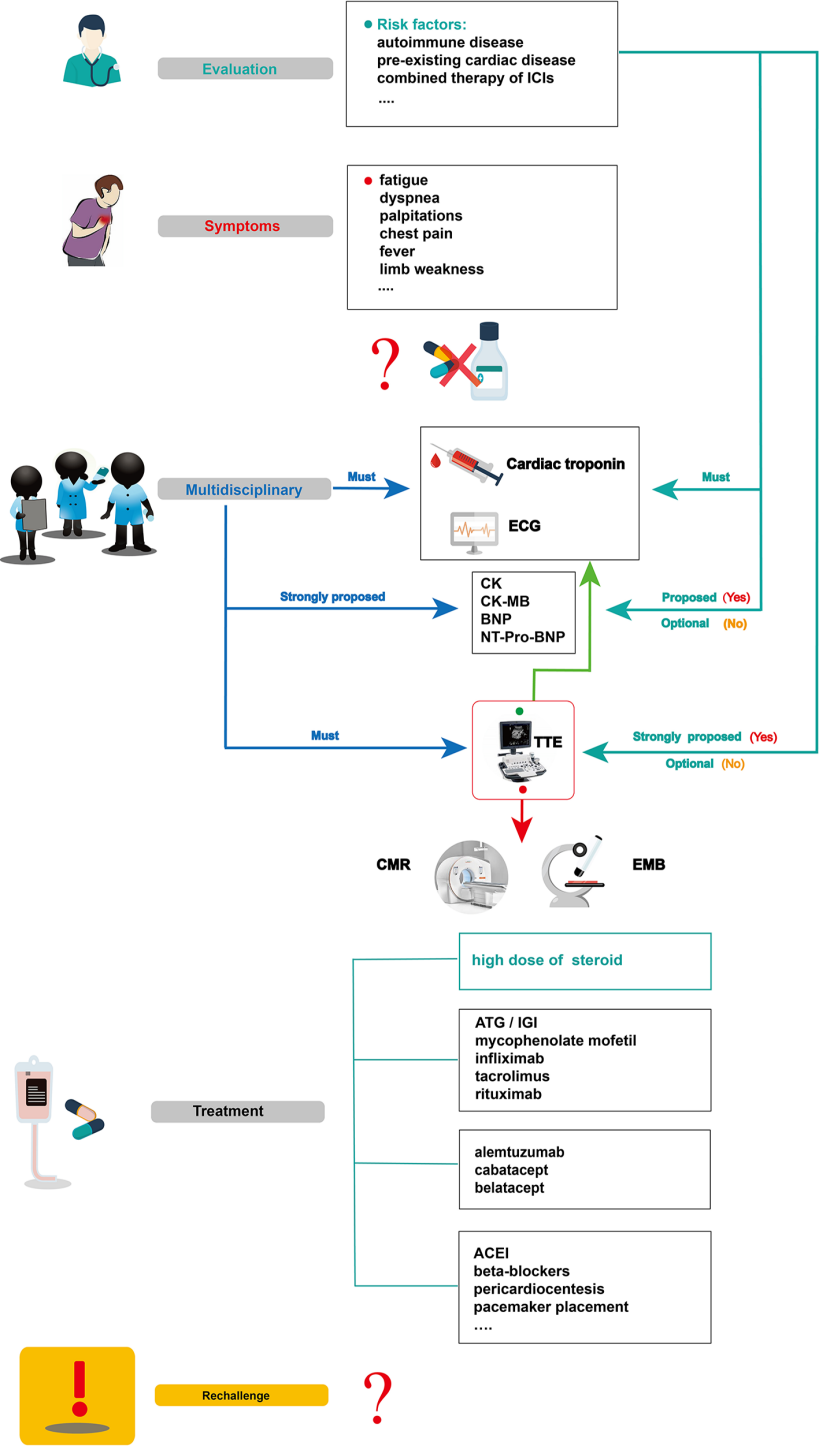
Summarized overview on potential monitoring and management of ICI-related cardiotoxicity. CK, creatine kinase; CK-MB, creatine kinase-myocardial band; BNP, brain natriuretic peptide; NT pro BNP, N-terminal pro-brain natriuretic peptide; TTE, transthoracic echocardiogram; CMR, cardiovascular magnetic resonance; EMB, endomyocardial biopsy; ATG, anti-thymocyte globulin; IGI, immunoglobulin; ACEI, angiotensin converting enzyme inhibitor.

Cardiotoxicity, a rare but fatal irAE, is particularly difficult to supervise and manage (Puzanov et al., 2017; Rapoport et al., 2017; Brahmer et al., 2018). Currently, no standard management guidelines on ICI-related cardiotoxicity have been established, due to its low incidence and the limited data on its manifestation, diagnosis, therapy, and outcomes. Based on previous studies and evidence, we summarized the possible management of ICI-related cardiotoxic reactions (**Figure 4**) as follows.

Before initiating ICI therapy, a comprehensive assessment of cardiovascular risk factors and a detailed cardiac history should be obtained for all patients, given the risk factors for ICI-associated cardiotoxicity remain unclear to date. According to previous reports, potential risk factors such as autoimmune disease, pre-existing cardiac disease, the combination of ICIs, and age should be evaluated before ICI treatment. In 50% of patients treated with ipilimumab, pre-existing autoimmune diseases worsened (Bowyer et al., 2016). Several studies have also revealed that patients with underlying autoimmune disease might be at high risk of irAEs, including cardiotoxicity (Johnson et al., 2016b; Johnson et al., 2017; Postow et al., 2018). For instance, patients with systemic autoimmune disorders are more likely to have subclinical myocarditis than those without autoimmune diseases. These results show that clinical and subclinical autoimmune diseases are an important consideration before initiation of ICI therapy (Varricchi et al., 2017). Physicians should be aware of potentially cardiotoxic events during ICI treatment, especially for those with pre-existing cardiac conditions (Hsu et al., 2018). In Heinzerling’s report, pre-existing cardiac disease or peripheral arterial disease had been present in most patients (5 of 8) who developed autoimmune cardiotoxicity (Heinzerling et al., 2016). The combination therapy of ICIs might also be a risk factor for ICI-associated myocarditis (Varricchi et al., 2017). Some VEGF inhibitors can increase the risk of thrombosis and coronary ischemia (Jain et al., 2018). They are also known to be related to cardiotoxicity and left ventricular dysfunction (Shah and Morganroth, 2015). Therefore, when ICIs are combined with anti-VEGF therapies, we should be vigilant for cardiac adverse effects. Given that the common adverse reaction to anti-VEGF therapies, such as axitinib, is hypertension (Hamnvik et al., 2015), it is not a likely causal agent in case of myocarditis; however, it can result in poor heart function in reaction to the physiological challenge. In addition, age can be associated with the incidence of ICI-associated cardiotoxicity. In Wang’s retrospective cohort, fatal irAEs involving cardiovascular toxicities were more common in the elderly than in the young (median,70 *vs* 62 years, *P* = .009) (Wang et al., 2018). Other risk factors, such as diabetes mellitus, a history of smoking, and dyslipidemia, might be included (Behling et al., 2017; Norwood et al., 2017; Tomita et al., 2017; Katsume et al., 2018; Agrawal et al., 2019; Altan et al., 2019), however, large sample studies are needed for confirmation.

To accurately diagnose ICI cardiotoxicity, several appropriate steps can be taken into consideration: First, serum troponin tests, baseline ECG, and serial surveillance are necessary. TTE and other laboratory tests, such as CK, CK-MB, BNP, or NT-proBNP can also be performed. Notably, for some special cases with a high risk of cardiotoxicity, TTE is strongly recommended to record the patient’s baseline cardiac function before the initiation of ICIs, and other laboratory inspections should be performed to record their baseline cardiac status. When a cardiotoxic reaction is suspected during treatment with ICIs alone and in combination—for example, the patient’s symptoms might include chest pain, dyspnea, fatigue, palpitations, fever, and limb weakness—one major challenge for oncologists is to identify whether these symptoms are clinical manifestations of cardiovascular irAEs. In a highly suspected ICI-associated cardiotoxic event, temporary discontinuation of ICIs is suggested; it is then necessary for the oncologists, cardiologists, and immunologists to together discuss further diagnosis and treatment. Second, apart from cardiac troponin and ECG, other laboratory biomarker tests, such as serum CK, CK-MB, BNP, and NT-proBNP tests, are strongly proposed. Although a TTE must be performed to record the heart function, normal cardiac function does not rule out ICI-induced myocarditis. CMR is also advisable to further evaluate abnormal cardiac structure. When CMR is not available or contraindicated, cardiac positron emission tomography/computed tomography is beneficial to diagnose myocardial inflammation. Third, in uncertain cases, EMB should be performed and samples from multiple sites should be collected to optimize the diagnostic accuracy of focal myocarditis and reduce sampling errors (Leone et al., 2012). After a comprehensive diagnosis, if there is no evidence of cardiac dysfunction, myocarditis, or other cardiotoxic events, ICI therapy can be slowly reintroduced under close troponin and ECG monitoring.

For patients with confirmed ICI-associated myocarditis, permanent discontinuation of ICIs after cardiac adverse events (G1) has been recommended by the American Society of Clinical Oncology guidelines, though the recommendation is based on anecdotal evidence (Brahmer et al., 2018). Then, prompt administration of oral prednisone (1–2 mg/kg/day) (Jain et al., 2017) or intravenous methylprednisolone (1–2 mg/kg/day) (Puzanov et al., 2017; Wang et al., 2017; Brahmer et al., 2018; De Almeida et al., 2018) should be initiated. If improved signs are observed, a slow tapering dose of glucocorticoid over at least 4 weeks has been recommended (Hu et al., 2019). In patients with pericarditis, despite the resolution of pericardial effusion *via* pericardial window, prednisone (1 mg/kg) initiation approximately 2 weeks later can prevent constrictive pericarditis (De Almeida et al., 2018). For patients with sick sinus syndrome, a low dose of cortisone (12.5 mg/day) taken orally might help relieve symptoms (Hsu et al., 2018). Nonetheless, if a patient shows a poor response to glucocorticoids, secondary drugs, including ATG, immunoglobulin, infliximab, tacrolimus, mycophenolate mofetil, rituximab, CTLA-4 agonists, and alemtuzumab can be considered (Jain et al., 2017; Norwood et al., 2017; Reddy et al., 2017; Frigeri et al., 2018; Esfahani et al., 2019; Salem et al., 2019). What should be emphasized is that infliximab is generally contraindicated because it can induce congestive heart failure (Kwon et al., 2003). Although the safety and efficacy of these immunosuppressive agents (*e.g.*, ATG, alemtuzumab, abatacept, belatacept) need further confirmation, these drugs could be a viable choice, especially for a critically ill patient with rapidly deteriorating cardiovascular function when high-dose glucocorticoid therapy is not possible.

In parallel with the immunosuppressive agents above, guideline-based therapy and supportive care is recommended for patients with ICI-associated cardiotoxicity. Patients with congestive heart failure should be treated with tolerable medications, including renin-angiotensin system inhibitors and beta-blockers (Yancy et al., 2017). Those with progressive life-threatening arrhythmias should be treated with appropriate antiarrhythmic drugs, or a patient with advanced conduction disease should be considered for temporary/permanent pacemaker placement. When necessary, invasive therapies such as pericardial window placement or pericardiocentesis might also be needed (Yang and Asnani, 2018).

It is controversial whether immunotherapy should be reintroduced after recovery from cardiac toxicity (Brahmer et al., 2018). Although cardiac dysfunction can be significantly improved by high-dose glucocorticoid therapy, an anti-PD1 antibody rechallenge might aggravate immune-related toxicity (Tajmir-Riahi et al., 2018). Given the potential for fulminant or fatal ICI-related myocarditis, ICIs are not recommended for reintroduction in patients (Champiat et al., 2016). However, in a retrospective analysis of 30 patients who were diagnosed with cardiotoxic irAEs, four patients resumed ICIs safely, without cardiotoxic event recurrence (Escudier et al., 2017).Therefore, the clinical oncologist, cardiologist, and immunologist collaboration should give discreet consideration to patients according to their manifestations, outcome, and alternative cancer treatment options to determine the safety of reintroducing ICI therapy.

In summary, the detection and management of ICI-associated cardiotoxic reactions are challenging, and more efforts are needed in future. First and foremost, one of the most important challenges is to improve preventive measures and increase early detection of cardiac toxicity *via* monitoring of cardiac damage. Second, a multidisciplinary team constituting of oncologists, cardiologists, radiologists, immunologists, and pathologists should be organized to achieve optimal management of ICI-induced cardiotoxicity and to decrease its lethal capacity. Further, developing cardiac protectants that can be used in conjunction with ICIs will be critical in preventing ICI cardiotoxicity. Last but not least, research into new immunotherapeutic agents with unknown cardiotoxicity incidence, such as anti-T cell Ig or anti-lymphocyte-activated gene-3 and mucin-containing protein 3, as well as V-domain Ig suppressor of T cell activation or B and T lymphocyte attenuator blockade, would be a new challenge for physicians. In addition, more clinical trials should focus on the effects of T cell costimulation blockers on cardiovascular disease. For example, CD40–TRAF6 inhibitors have already been well examined. Blocking OX40 and anti-4-1BB costimulation could be a promising strategy in the future (Simons et al., 2019).

## Conclusion

Immune checkpoint inhibitors, either alone or in combination, can result in cardiotoxic adverse reactions, such as myocarditis, pericarditis, conduction abnormalities, cardiomyopathy, acute coronary syndrome, and others. Of all ICI-related cardiotoxic events, myocarditis is the most common cardiotoxic reaction. Though the incidence of ICI-associated cardiotoxicity remains relatively low, clinicians must be aware of these adverse events due to their high fatality rate. It mainly occurs in the early stage after ICI initiation, with nonspecific symptoms ranging from asymptomatic cardiac biomarker elevation, fatigue, and general malaise to chest pain, dyspnea, palpitations, multiorgan failure, cardiogenic shock, and cardiac arrest. A high level of clinical suspicion and early diagnosis indicators are required due to the rapid progress and fulminant course of the disease. The assessment of clinical features in combination with laboratory examinations (cTnI, cTnT, CK, CK-MB, BNP, and NT-proBNP), ECG, TTE, CMR, and EMB contribute to the diagnosis of ICI-associated cardiotoxicity. Among these diagnostic methods, troponin is generally the most sensitive marker, ECG has widespread availability and is easily performed, and EMB is a gold standard diagnosis. Before initiating ICIs, a comprehensive assessment of cardiovascular risk factors and a detailed cardiac history should be obtained, especially for patients with autoimmune disease or pre-existing cardiac disease, and when ICIs are combined with other treatments. For patients with confirmed cardiotoxic events, prompt high-dose steroids and other immunosuppressors, such as ATG, immunoglobulin, infliximab, tacrolimus, mycophenolate mofetil, rituximab, CTLA-4 agonists, and alemtuzumab can result in clinical recovery and increased survival. Auxiliary therapies, such as ACEIs, beta-blockers, aspirin, diuretics, antiarrhythmic drugs, pacemaker placement, and pericardiocentesis can also help. In addition, cardiac function assessment and frequent monitoring are necessary. To better understand the pathogenesis of this disease and provide effective treatment strategies, larger studies are needed.

## Author Contributions

YZhou collected and reviewed the literature and wrote the manuscript. YZhu wrote and revised the manuscript. MW and YX rechecked the manuscript and put forward meaningful comments on it. CC, TZ, and FX assisted in drawing. JL and ZD contributed equally to writing the design and revised the manuscript. All authors read and approved the final manuscript.

## Funding

This study was supported by the National Clinical Research Center for Geriatrics (West China Hospital, Z2018B12),1.3.5 Project for Disciplines of Excellence, West China Hospital, Sichuan University (ZYJC18022), and Sichuan Science and Technology Department Key Research and Development Project (2019YFS0539).

## Conflict of Interest

The authors declare that the research was conducted in the absence of any commercial or financial relationships that could be construed as a potential conflict of interest.
